# Supplementary material for: Management and Clinical Outcomes of Breast Cancer in Women Diagnosed with Hereditary Cancer Syndromes in a Clinic-Based Sample from Colombia
Source: Cancers (Basel). 2024 May 26;16(11):2020. doi: 10.3390/cancers16112020 (PMC11171067; doi:10.3390/cancers16112020)
Supplement: Supplementary file 1 [file cancers-16-02020-s001.zip › cancers-2969702-supplementary.pdf]

Supplementary Table

Supplementary Table S1. Genes included in the multigene panel

AIP	*ALK*	*APC*	*ATM*	*BAP1*	*BARD1*	*BLM*	*BMPR1A*
BRCA1	*BRCA2*	*BRIP1*	*BUB1B*	*CASR*	*CDC73*	*CDH1*	*CDK4*
CDKN1B	*CDKN1C*	*CDKN2A*	*CEBPA*	*CEP57*	*CHEK2*	*CYLD*	*DDB2*
DICER1	*DIS3L2*	*EGFR*	*EPCAM*	*ERCC2*	*ERCC3*	*ERCC4*	*ERCC5*
EXT1	*EXT2*	*EZH2*	*FANCA*	*FANCB*	*FANCC*	*FANCD2*	*FANCE*
FANCF	*FANCG*	*FANCI*	*FANCL*	*FANCM*	*FH*	*FLCN*	*GATA2*
GNAS	*GPC3*	*HNF1A*	*HRAS*	*KIT*	*MAX*	*MEN1*	*MET*
MLH1	*MRE11A*	*MSH2*	*MSH6*	*MUTYH*	*NBN*	*NF1*	*NF2*
NSD1	*PALB2*	*PDE4D*	*PHOX2B*	*PMS1*	*PMS2*	*POLD1*	*POLE*
PPM1D	*PRF1*	*PRKAR1A*	*PTCH1*	*PTEN*	*RAD50*	*RAD51C*	*RAD51D*
RB1	*RECQL4*	*RET*	*RHBDF2*	*RUNX1*	*SBDS*	*SDHA*	*SDHAF2*
SDHB	*SDHC*	*SDHD*	*SLX4*	*SMAD4*	*SMARCB1*	*STK11*	*SUFU*
TMEM127	*TP53*	*TSC1*	*TSC2*	*VHL*	*WRN*	*WT1*	*XPA*
XPC							
